# Genome-Wide Identification, Characterization, and Expression Analysis of the Grapevine Superoxide Dismutase (SOD) Family

**DOI:** 10.1155/2019/7350414

**Published:** 2019-02-24

**Authors:** Xiaoxuan Hu, Chenyu Hao, Zong-Ming Cheng, Yan Zhong

**Affiliations:** College of Horticulture, Nanjing Agricultural University, Nanjing 210095, China

## Abstract

Superoxide dismutase (SOD) is an essential enzyme of the plant antioxidant system that responds to oxidative damage caused by adverse conditions. However, little is known about the SOD gene family in *Vitis vinifera* (Vv). In the present study, ten SOD genes, including 6 copper/zinc SODs, 2 iron SODs, and 2 manganese SODs, were identified in the grapevine genome where they were unevenly distributed on 12 chromosomes. Ten VvSOD genes were divided into three main groups based on phylogenetic analysis, subcellular localization, and the distribution of conserved protein motifs. Additionally, many *cis*-elements related to different stresses were found in the promoters of the 10 VvSOD genes. Syntenic analysis revealed that *VvMSD1* and *VvMSD2* were derived from segmental duplication, and *VvCSD4* and *VvCSD5* belong to a pair of tandemly duplicated genes. Gene expression analysis based on microarray data showed that the 10 VvSOD genes were expressed in all the tested tissues. Interestingly, the segmentally duplicated gene pair (*VvMSD1* and *VvMSD2*) exhibited differential expression patterns in various organs. In contrast, the tandemly duplicated gene pair (*VvCSD4* and *VvCSD5*) displayed similar expression patterns in the tested organs. Our results provide a basis for further functional research on the SOD gene family in grapevine.

## 1. Introduction

Grapevine (*Vitis vinifera*) is one of the most cultivated and economically valuable fruit crops in the world. However, abiotic stresses, particularly drought and salt stress, threaten the growth of grapevine globally, thereby affecting fruit yield and quality [1–3]. The most common result of such stress is the generation of toxic reactive oxygen species (ROS). Membrane damage, protein oxidation, DNA lesions, and even irreparable metabolic dysfunction and cell death can occur as a result of excessive ROS (such as superoxide anions, hydroxyl radicals, hydrogen peroxide, and singlet oxygen) [4, 5].

To cope with ROS toxicity, plants have developed efficient and sophisticated antioxidative response systems, including the synthesis of low relative molecular mass antioxidant molecules (e.g., l-ascorbic acid and glutathione) and various enzymes. Among these enzymatic components, the superoxide dismutases (SODs) constitute the first line of defense against ROS by catalyzing the dismutation of the superoxide O_2_^−^to O_2_ and H_2_O_2_ [6, 7]. In plants, SODs have been detected in the roots, leaves, fruits, and seeds where they play important roles in protecting cells from oxidative damage [8].

Multiple SOD isozymes exist in plants and are classified into three types based on their metal cofactor, protein folds, and subcellular distribution: copper/zinc- (Cu/Zn-) SOD, manganese- (Mn-) SOD, and iron- (Fe-) SOD, and these SODs are located in different compartments of the cell [7, 9, 10]. Cu/Zn-SODs are mainly distributed in the cytosol, chloroplasts, peroxisomes, and/or the extracellular space, while Fe-SODs mainly occur in the chloroplasts, as well as in peroxisomes and mitochondria, and Mn-SODs are mainly localized not only in the mitochondria but also in different types of peroxisomes [11]. In addition, a new type of SOD, nickel (Ni)SOD, was first discovered, cloned, and characterized in *Streptomyces* [12]. However, no evidence for NiSOD has been found in plants [13].

In recent years, some studies have reported that SODs contribute to the response to various environmental stimuli in plants, such as cold, drought, salinity, auxin, and ethylene [14–16]. Owing to their crucial roles in the antioxidant system, a considerable number of SOD genes have been cloned from various monocot and dicot plants [17–19]. With the development of high-throughput sequencing technologies, the genome-wide identification of SOD gene families has been performed in many plant species, including *Arabidopsis thaliana* [20], *Dimocarpus longan* [17], *Sorghum bicolor* [21], *Populus trichocarpa* [9], *Musa acuminate* [22], *Gossypium raimondii*, *Gossypium arboreum* [23], and *Gossypium hirsutum* [24].

In the present study, we identified the SOD gene family in grapevine on the genome-wide scale. Genomic organization, gene structure, motif composition, subcellular localization, syntenic analysis, and phylogenetic relationships were analyzed using bioinformatics. Then, the gene ontology and putative promoters of the grapevine SODs were also investigated, and the *cis*-elements involved in stress responses were analyzed. Putative transcription factors (TFs) which may regulate the VvSOD genes were predicted. Furthermore, we studied the expression patterns of the grapevine SOD gene family under abiotic stress (salt, drought, cold, and heat) using microarray data and a real-time quantitative- (qRT-) PCR detection system. Finally, coexpression networks of VvSOD genes and TF genes under the four abiotic stresses were generated based on the qRT-PCR data. This was the first comprehensive study of the SOD gene family in grapevine and may provide valuable information for understanding the classification, evolution, and putative functions of the grapevine SOD family on the genome-wide scale.

## 2. Materials and Methods

We downloaded the most recent version (V 2.1) of a 12X assembly of the grapevine (*V. vinifera*) genome from CRIBI (http://genomes.cribi.unipd.it/grapevine/) for identifying SOD genes. To identify members of the SOD gene family in grapevine, the full-length protein sequences from *A. thaliana* (TAIR locus number: AT1G08830.1, AT2G28190, AT5G18100, AT4G25100, AT5G51100, AT5G23310, AT3G56350, and AT3G10920) were used as BLASTp queries against all the grapevine protein sequences with the threshold expectation value set to 1.0. All the hits were further submitted to Pfam analysis (http://pfam.xfam.org/) to verify the presence of the SOD domain. Physicochemical characteristics of the SOD proteins were calculated using the ProtParam tool (http://web.expasy.org/protparam/), including the number of amino acids, molecular weight, theoretical isoelectric point (pI), aliphatic index, and grand average of hydropathicity (GRAVY) [25].

ProtComp9.0 server (http://linux1.softberry.com/) was used to predict the subcellular localizations of the SOD proteins [22]. Conserved protein motifs were predicted by MEME Suite (http://meme-suite.org/tools/meme) with the default settings, except that the number of motifs was set to 8, and the minimum and maximum motif widths were set to 20 and 150 amino acids [26]. The web-based Gene Structure Display Server (GSDS; http://gsds.cbi.pku.edu.cn/index.php) program was utilized to illustrate exon and intron organization for the grapevine SOD genes.

### 2.1. Phylogenetic Analysis

To investigate the phylogenetic relationships of the SODs among grapevine and *A. thaliana*, multiple SOD protein sequences were aligned, and an unrooted phylogenetic tree was constructed in MEGA 6.06 [27]. The phylogenetic tree was constructed using the neighbor-joining (NJ) method and Jones-Taylor-Thornton (JTT) model. In the phylogenetic tree, the degree of support for a grouping pattern was evaluated using bootstraps (1000 replicates).

### 2.2. Chromosomal Locations and Syntenic Analysis

The chromosomal locations of the grapevine SOD genes were verified from the CRIBI database (http://genomes.cribi.unipd.it/grapevine/), and chromosomal images were drawn using MapInspect software [28].

Duplication patterns of the VvSOD genes were assigned based on their locations. Genes located in a 200 kb region of a chromosome or a scaffold were defined as a gene cluster derived from tandem duplications [29]. Segmentally duplicated genes were identified as genes located on duplicated chromosomal blocks and were determined using MCScanX software (http://chibba.pgml.uga.edu/mcscan2/), which detects gene duplication events using an *E*-value threshold of 10^−5^ [30].

To detect the selection mode of the segmentally duplicated gene pairs, the ratio of nonsynonymous substitutions to synonymous substitutions was evaluated. Firstly, the CDSs (nucleotide coding sequences) of the VvSOD genes were aligned using Muscle in MEGA6.06. Then, nonsynonymous substitutions (Ka), synonymous substitutions (Ks), and the ratio between them (Ka/Ks) were calculated using MEGA 6.06.

### 2.3. GO Analysis and Promoter Sequence Analysis

The gene ontology (GO) term IDs of each VvSOD gene member in grapevine were obtained from the CRIBI database (http://genomes.cribi.unipd.it/DATA/V2/annotation/bl2go.annot.txt). The annotations of the GO term IDs were collected from the Gene Ontology Consortium (http://www.geneontology.org). Ten grapevine SOD proteins were assessed based on their molecular functions, biological processes, and cellular localizations.

The 2000 bp upstream sequences of the coding region of each VvSOD gene were downloaded from the CRIBI database. Then, the *cis*-elements in the promoters of each VvSOD gene were predicted using the PlantCARE server (http://bioinformatics.psb.ugent.be/webtools/plantcare/html/) [31].

### 2.4. Prediction of Potential Regulatory Interactions between TFs and VvSODs

The TFs which possess overrepresented targets in the VvSOD gene sets were detected from the Plant Transcriptional Regulatory Map (PlantRegMap) with a regulation prediction tool (http://plantregmap.cbi.pku.edu.cn/regulation_prediction.php) [32]. 2000 bp upstream sequences of the coding region of each VvSOD gene were used as the input.

### 2.5. Microarray Data Analysis

Microarray gene expression profiles covering most of the grapevine organs at different stages were downloaded from Gene Expression Omnibus (GEO, available online: https://www.ncbi.nlm.nih.gov/geo/) under the accession number GSE36128 [33]. The collected plant organs were as follows: bud, inflorescence, tendril, leaf, stem, root, developing berry, withering berry, seed, rachis, anther, carpel, petal, pollen, and seedling.

Expression analyses of SOD genes and TF genes in response to abiotic stresses were based on microarray data (including accession number GSE31594 and GSE31675) downloaded from the NCBI GEO datasets. Four abiotic stresses, including cold, salt, heat, and polyethylene glycol (PEG), were analyzed. The fold changes compared with the corresponding control in each experiment were used to generate heatmaps using the R package (https://www.r-project.org/) pheatmap.

### 2.6. Coexpression Network and Interaction of VvSODs and TF Genes

For the four stress treatments, the Pearson correlation coefficient (PCC) value (Table S4) was calculated between each pair of VvSODs and TF genes using gene expression values from microarray data in SPSS v 20.0 (IBM Corp., Armonk, NY, US) [28]. Coexpressed gene pairs were filtered with a PCC cutoff of 0.95. The coexpression network map was generated by Cytoscape 3.3 (http://www.cytoscape.org).

### 2.7. Plant Material and Experimental Treatments

For the cold, heat, and salt treatments, six-week-old “Pinot Noir” grapevine (*V. vinifera*, the sequenced genotype PN40024) plantlets were used in this study. The plantlets were maintained at a photoperiod of 16 h light/8 h dark and a temperature of 23°C. Low temperature and heat stress treatments were applied by placing the plants at 4°C (drug storage box, HYC-360, Haier) and 42°C (intelligent artificial climate box, RXZ-380C, Jiangnan Instrument Factory, Ningbo) with a photoperiod of 16 h light/8 h dark, respectively [28]. Leaf samples were harvested from both treatments at 0, 6, 12, and 24 h after initiation of the treatments. Plants subjected to 200 mmol/L NaCl were sampled at 0, 6, 12, and 24 h after treatment. Three independent biological replications were performed for each treatment. Tissue samples were immediately frozen in liquid nitrogen and stored at −70°C until analysis.

For drought stress treatment, *in vitro*-grown grapevine plants (PN40024) were maintained on half-strength Murashige and Skoog medium containing 0.3 mg/L indole 3-butyric acid (IBA) and placed at 25°C in a culture room under a photoperiod of 16/8 h and a light intensity of 100 *μ*mol·m^−2^·s^−1^. Five-week-old tissue-cultured grapevine plants were transplanted into pots and acclimatized in the growth chamber with 16 h light at 24°C/8 h dark at 22°C and 70%–80% relative humidity [34]. Plants grown in pots were fully watered for the first four weeks, after which water was withheld to impose the drought treatment. The grapevine leaf samples were collected at 0 (control), 2, 4, and 8 days after the treatment, respectively.

### 2.8. Total RNA Extraction and cDNA Synthesis

Total RNA was extracted from the leaves using the Plant Total RNA Isolation Kit Plus. The total RNA was eluted in 30 *μ*L of RNase-free water and stored at −70°C. The purity and density of the extracted RNA was assessed on a NanoDrop ND-1000 spectrophotometer (NanoDrop Technologies, USA) using RNase-free water as a blank.

Single-stranded cDNA was synthetized using a Takara PrimeScript RT reagent kit with a gDNA eraser (Takara, Dalian, China) and oligo dT primers as described by the manufacturer's protocol. Approximately 2 *μ*g of total RNA in a single 20 *μ*L reaction was converted to single-stranded cDNA using standard thermal cycling conditions.

### 2.9. qRT-PCR Analysis of VvSOD Genes

qRT-PCR analyses were conducted on a Roche LightCycler 480 PCR system using Lightcycler 480 SYBR Green I Master. The oligonucleotide primers (Table S3) for amplifying specific VvSODs were designed using the PrimerQuest Tool (http://sg.idtdna.com/Primerquest/Home/Index). The 10 *μ*L reaction volume contained 5 *μ*L SYBR Green I Master, 1 *μ*L of diluted cDNA, 1 *μ*L of each primer (10 *μ*M), and the addition of ddH_2_O to bring the total volume to 10 *μ*L. The thermocycle protocol was as follows: denaturation at 95°C for 10 min, followed by 40 cycles of 95°C for 10 s, 58°C for 20 s, and 72°C for 10 s. At the end, a melting curve was generated from 65 to 97°C. Three replicates were used for each sample. Expression levels were calculated using the 2^-ΔΔT^ method and the housekeeping gene (actin-101-like, *VIT_012s0178g00200*) was used as a reference gene for normalizing the expression of the VvSOD genes [35]. Fold differences were visualized by normalizing all of the data based on setting the expression level at 0 h as a value of 1 (values above 1 and below 1 were considered as up- and downregulated, respectively) [36].

## 3. Results

### 3.1. Genome-Wide Identification of the SOD Gene Family in Grapevine

Based on a BLAST search using the known *A. thaliana* SOD protein sequences and the identification by the Pfam database, a total of 10 SOD genes were identified in the grapevine genome. Based on the domain analysis, the 10 grapevine SOD genes were classified into 3 groups (Cu/ZnSODs, FeSODs, and MnSODs), including 6 VvSOD proteins with a copper-zinc domain (Pfam: 00080) and 4 SOD proteins with an iron/manganese superoxide dismutase alpha-hairpin domain (Pfam: 00081) or an iron/manganese superoxide dismutase, C-terminal domain (Pfam: 02777). Based on the domain analysis and chromosome location, the 10 grapevine genes were named as follows: *VvCSD1*, *VvCSD2*, *VvCSD3*, *VvCSD4*, *VvCSD5*, *VvCSD6*, *VvMSD1*, *VvMSD2*, *VvFSD1*, and *VvFSD2* (Table 1).

The physicochemical analysis indicated that the length of the amino acid sequences, MWs, pIs, aliphatic indexes, and GRAVY values varied among these VvSOD proteins. Considerable variations in the number of amino acids among these VvSOD proteins were observed, ranging from 79 to 329 aa. The predicted molecular weight of the 10 VvSOD proteins varied from 8594.7 to 37779.23 Da. The results revealed that four Fe-MnSODs were basic and five Cu/ZnSODs were acidic, except for the slightly basic VvCSD3. The GRAVY numeric values of the three VvSOD proteins (*VvCSD1*, *VvCSD2*, and *VvCSD5*) were positive, indicating that they are hydrophobic proteins and the rest of the VvSODs are hydrophilic proteins (Table 1).

The ProtComp9.0 program was used to predict the subcellular localizations of the VvSOD proteins. Among them, six Cu/ZnSODs were cytoplasmic, two MnSODs were in the mitochondria, and two FeSODs were located on the chloroplast (Table 1). These results were in accordance with a previous study [8, 37].

**Figure 1:**
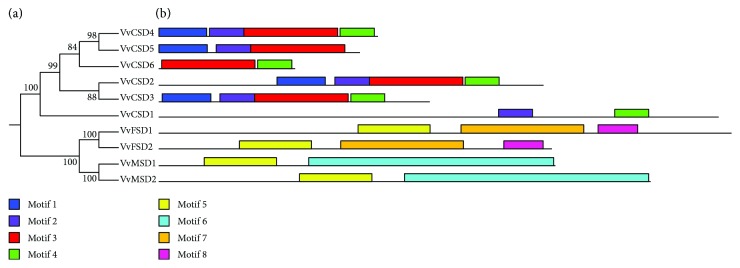
Unrooted neighbor-joining phylogenetic tree and conserved motif analysis of VvSOD proteins. (a) The phylogenetic tree was generated based on the protein sequences of VvSOD proteins. (b) Conserved motif analysis of VvSOD proteins. Different color boxes represent different types of motifs.

**Figure 2:**
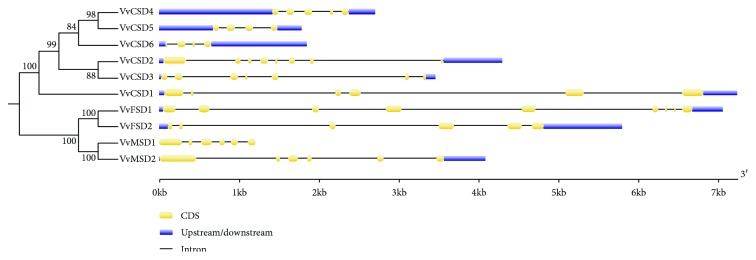
Gene structure of VvSOD genes. Blue boxes represent exons and lines represent introns.

The MEME server was used for conserved motif analysis, and a total of eight conserved motifs were identified (Figure 1). Among them, motif 3 was observed in all six Cu/ZnSODs, while motifs 5, 6, and 7 were found in four Fe-MnSODs (Figure 2). The exon numbers varied among the 10 VvSODs, ranging from 4 to 9 (Figure 2). It is worth noting that the tandemly duplicated gene pair (*VvCSD4* and *VvCSD5*) and segmentally duplicated gene pair (*VvMSD1* and *VvMSD2*) both exhibited the same exon numbers.

### 3.2. Phylogenetic Analysis of SOD Genes

**Figure 3:**
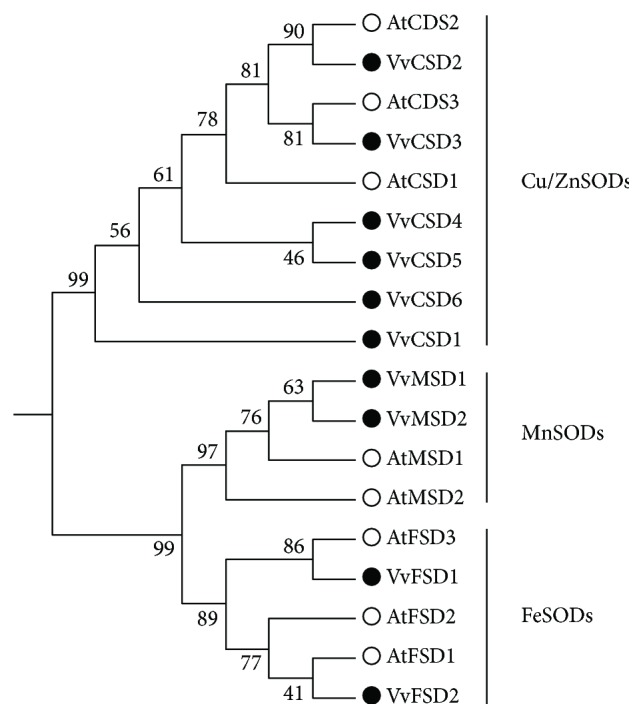
Phylogenetic tree of 77 SOD proteins from grape and other plants including *Arabidopsis thaliana*, *Oryza sativa*, *Sorghum bicolor*, *Populus trichocarpa*, *Amborella trichopoda*, *Cucumis sativus*, *Solanum lycopersicum*, and *Gossypium raimondii*. The Jones-Taylor-Thornton model with *P*-distance was chosen to conduct the phylogenetic tree. The bootstrap value was set as 1000. The SOD genes were classified into two major groups and five subfamilies (groups a, b, c, d, and e which have red, blue, yellow, purple, and green branches, respectively).

Twenty-eight SOD protein sequences from grapevine and *A. thaliana* (Table S1) were used to construct an unrooted phylogenetic tree (Figure 3). The SODs were divided into three major groups, namely, Cu/ZnSODs, MnSODs, and FeSODs. The FeSODs and MnSODs were clustered within a large clade with a high bootstrap value (99%), implying that the FeSODs and MnSODs originated from a common ancestor. Additionally, species-specific duplications occurred between *VvCSD4* and *VvCSD5* and *VvMSD1* and *VvMSD2*.

### 3.3. Chromosomal Locations and Syntenic Analysis

**Figure 4:**
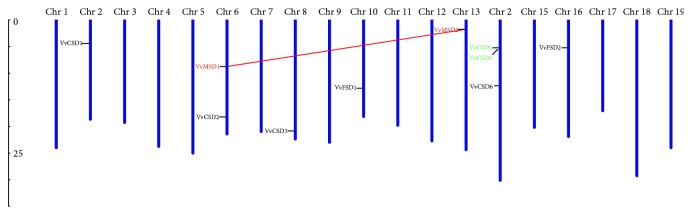
Chromosomal locations of 10 VvSOD genes on grape chromosomes. The chromosome numbers are indicated at the top of chromosomes, and the size of chromosomes is represented with a vertical scale. Segmental duplication relationships and DNA-based transposed duplication relationships among SOD genes are indicated in red and green, respectively.

The 10 VvSOD genes were located on seven chromosomes, including *VvCSD1* on chromosome 2; *VvCSD2* and *VvMSD1* on chromosome 6; *VvCSD4*, *VvCSD5*, and *VvCSD6* on chromosome 14; and *VvCSD3*, *VvFSD1*, *VvMSD2*, and *VvFSD2* on chromosomes 8, 10, 13, and 16, respectively (Figure 4). One segmental duplication event was detected between gene *VvMSD1* and *VvMSD2* on chromosomes 6 and 13. Moreover, *VvCSD4* and *VvCSD5* were identified as a pair of tandemly duplicated genes. The Ka/Ks ratios were calculated to better understand the duplication and functional divergence of the duplicated VvSOD genes during their evolutionary course. As shown in Table S2, the Ka/Ks ratio of the segmentally duplicated gene pair (*VvMSD1* and *VvMSD2*) was 0.29, indicating that they underwent purifying selection. The Ka/Ks ratio of the tandemly duplicated gene pair (*VvCSD4* and *VvCSD5*) was 1.29, which indicated that they were driven by positive selection during their evolutionary process.

### 3.4. GO Analysis of the VvSOD Proteins

**Figure 5:**
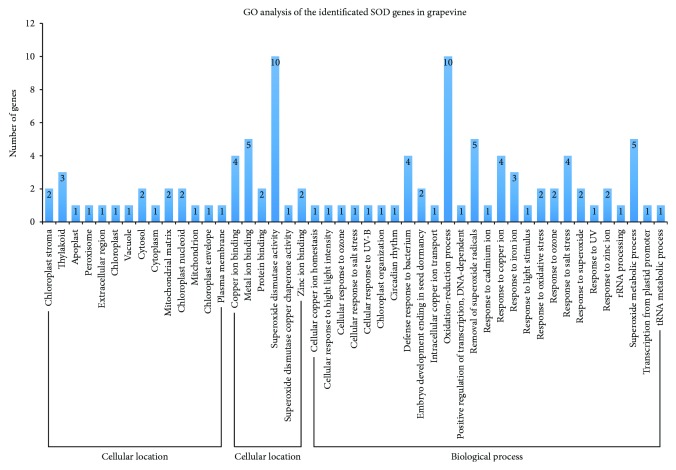
Gene ontology of SOD genes in grapevine. “Biological process,” “cellular location,” and “molecular function” were treated as independent attributes.

Biological processes, molecular functions, and cellular components of genes are characteristics of genes or gene products that elucidate the diverse molecular functions of proteins [38]. GO analysis was performed to further characterize the predicted functions of the 10 VvSOD proteins (Figure 5). The “molecular function” data showed that all VvSOD genes were involved in the “superoxide dismutase activity” (GO:0004784). According to the “biological process” results, all of the VvSOD genes participated in the “oxidation-reduction process” (GO:0055114). Moreover, the grapevine SOD genes may be involved in the biological processes responding to biotic stimulus and abiotic stimulus, such as strong light, ozone, salt, and UV stresses. In addition, they may also participate in some biological metabolic processes, such as superoxide metabolic processes, tRNA metabolic processes, and rRNA processing.

### 3.5. Analysis of *cis*-Elements in Putative VvSOD Gene Promoters

**Figure 6:**
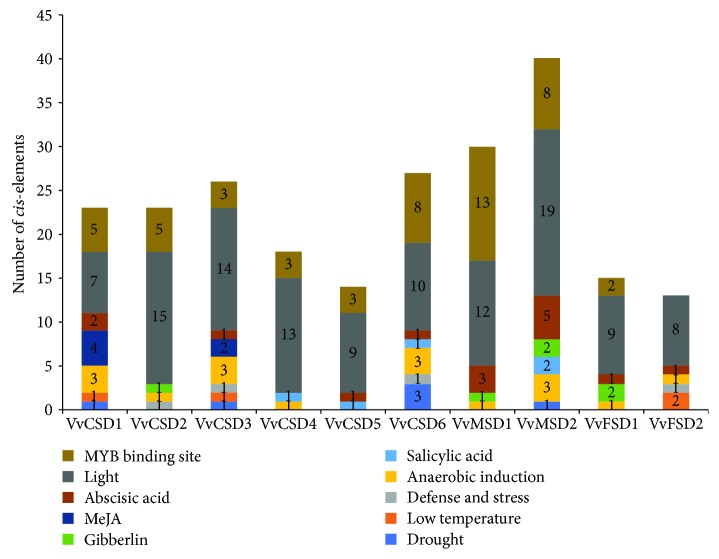
*cis*-element analysis of putative VvSOD promoters related to stress responses. Different *cis*-elements with the same or similar functions are shown in the same color.

To further determine the regulatory roles of VvSODs under various stresses, the *cis*-elements in the VvSOD gene promoter sequences were researched. The *cis*-elements were divided into four major subgroups: stress-responsive, hormone-responsive, light-responsive, and MYB binding site (Figure 6). Among 10 VvSODs, nine of them possessed several MYB binding sites. Furthermore, we found that abundant light-responsive *cis*-elements existed in the VvSODs, particularly in the *VvMSD2* gene with a number of 19.

### 3.6. Prediction of Potential Regulatory Interactions between TFs and VvSODs

To investigate the potential regulatory interactions between TFs and VvSODs, regulation prediction was done. A total of 21 TFs which possess overrepresented targets in the input gene set under a cutoff *p* value ≤ 0.05 were found. Among them, 10, 2, 9, 10, 9, 5, 1, 13, 3, and 8 TFs targeted *VvCSD1*, *VvCSD2*, *VvCSD3*, *VvCSD4*, *VvCSD5*, *VvCSD6*, *VvFSD1*, *VvFSD2*, *VvMSD1*, and *VvMSD2*, respectively (Table S6).

### 3.7. Expression Analysis of the VvSOD Genes Based on Microarray Data

**Figure 7:**
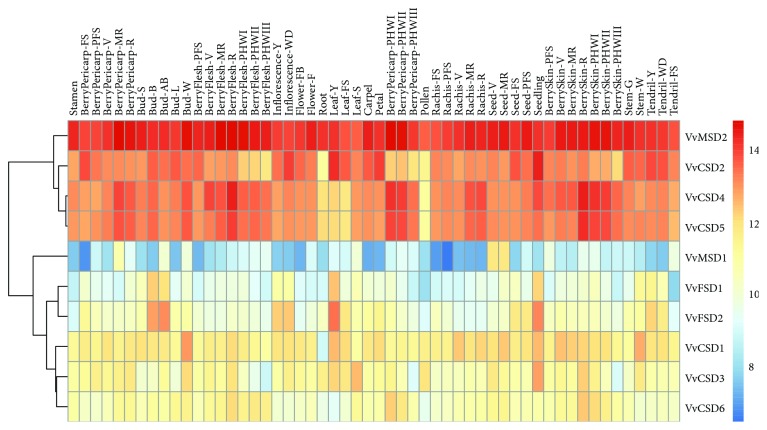
Expression profiles of 10 VvSODs in 54 different tissues and stages. Microarray data of the different organs of grapevine at various developmental stages were downloaded from Gene Expression Omnibus (GEO, available online: https://www.ncbi.nlm.nih.gov/geo/) under the accession number GSE36128, processing as log2 of the ratio and graphically represented with the RStudio software.

To gain more insight into the role that VvSODs play in plant growth and development, we analyzed the expression patterns of the VvSOD genes in different grapevine tissues and organs based on the microarray data from 54 grapevine samples [33]. Interestingly, *VvMSD2* displayed consistently high expression in all 54 tissues, whereas *VvMSD1* exhibited lower expression levels compared with the others (Figure 7). *VvCSD4* and *VvCSD5* exhibited similar expression patterns in all the tested tissues. This similarity was also evident in gene *VvFSD1* and *VvFSD2*. Additionally, two genes (*VvMSD1* and *VvMSD2*) demonstrated distinct tissue-specific expression patterns.

**Figure 8:**
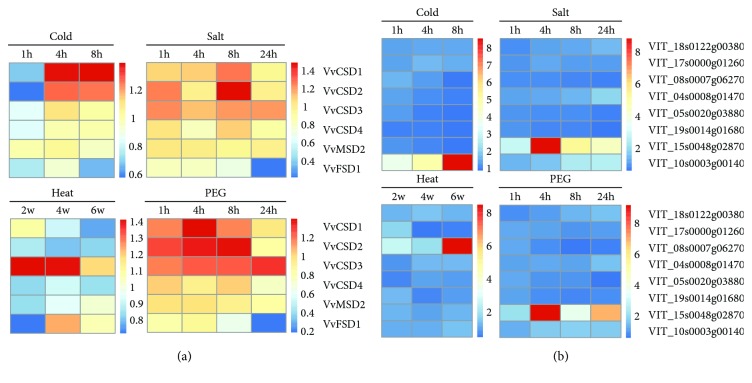
Expression profiles of the response of the grapevine VvSODs (a) and TF genes (b) to several abiotic stresses. Microarray data (accession number GSE31594 and GSE31675) downloaded from the NCBI GEO datasets. Four abiotic stresses, including salt, polyethylene glycol (PEG), heat, and cold, were analyzed. The fold changes compared with the corresponding control in each experiment were used to perform heatmaps that were generated by RStudio software.

Furthermore, four Affymetrix microarray datasets were specifically analyzed for determining the expression profiles of VvSODs and TF genes in response to abiotic stresses, which included PEG, salt, heat, and cold. Six out of 10 probes corresponding to VvSOD genes were found (*VvCSD1*, *VvCSD2*, *VvCSD3*, *VvCSD4*, *VvMSD2*, and *VvFSD1*). Eight out of 21 probes corresponding to TF genes were found (*VIT_18s0122g00380*, *VIT_17s0000g01260*, *VIT_08s0007g06270*, *VIT_04s0008g01470*, *VIT_05s0020g03880*, *VIT_19s0014g01680*, *VIT_15s0048g02870*, and *VIT_10s0003g00140*). In the cold, salt, and PEG stress, the expression levels of *VvCSD1* and *VvCSD2* were higher than those of the other four genes (Figure 8(a)). *VvFSD1* was expressed at an extremely low level in these three treatments. However, *VvCSD3* and *VvFSD1* were highly induced under the heat stress treatment. In each abiotic stress, there existed a single TF gene which responded strongly. However, the other was expressed at the similar level (Figure 8(b)). For example, listed genes, *VIT_10s0003g00140* in cold stress, *VIT_15s0048g02870* in salt and PEG stress, and *VIT_08s0007g06270* in heat stress, were all altered strongly by 8 times compared with the control. *VIT_10s0003g00140*, *VIT_15s0048g02870* and *VIT_08s0007g06270* belong to ERF, HD-ZIP, and SBP TF family, respectively (Table S6).

### 3.8. Expression Analysis of VvSOD Genes Based on qRT-PCR

**Figure 9:**
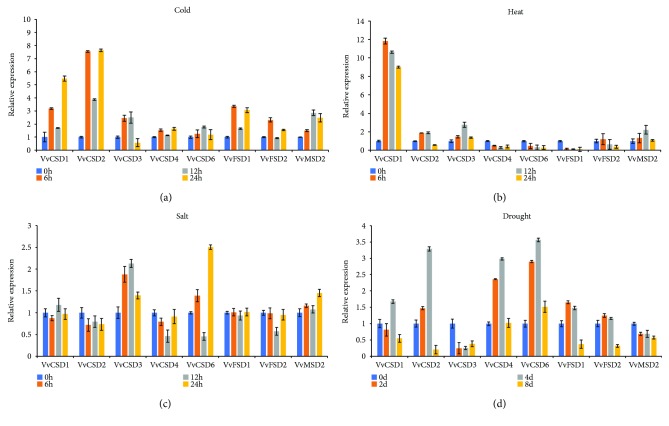
Quantitative real-time polymerase chain reaction (PCR) analysis of grape VvSODs in response to (a) cold stress, (b) heat stress, (c) salt stress (0 h, 6 h, 12 h, and 24 h), and (d) drought stress (0 days, 2 days, 4 days, and 8 days). Transcripts were normalized to the actin gene expression. The mean ± SD of the three biological replicates is presented.

Previous studies have demonstrated the important roles that SOD genes play in the plant response to abiotic stresses. The analysis of microarray data also indicated that the VvSOD genes were induced by different stresses. To gain more insight into the relative expressional diversity of eight members of the VvSOD gene family to cold, heat, salt, and drought, we detected the expression levels using qRT-PCR (Figure 9). Our results demonstrated that all eight VvSOD genes were induced noticeably by the four stresses, exhibiting different expression levels. All eight members were upregulated in the cold treatment, except for *VvCSD3* and *VvFSD2* (Figure 9). Among the six upregulated genes, *VvCSD1*, *VvCSD2*, and *VvFSD1* were altered strongly by 3–7.7 times compared with the control. Upon heat stress, expression of *VvCSD1* increased by 12-fold, 11-fold, and 9-fold at 6 h, 12 h, and 24 h, respectively. *VvCSD2*, *VvCSD3*, *VvFSD2*, and *VvMSD2* were also upregulated at 6 h with a lower expression level compared with that in *VvCSD1*. However, the other three genes (*VvCSD4*, *VvCSD6*, and *VvFSD1*) were downregulated during the treatment periods (Figure 9). In the salt treatment, the eight genes showed the lowest expression levels compared with the other three stresses. The expression levels of most of the genes exhibited slight variations, except *VvCSD3* and *VvCSD6*, which were upregulated more than 2-fold. Upon drought stress, the expression level of *VvCSD1*, *VvCSD2*, *VvCSD4*, and *VvCSD6* peaked at 12 h, but six genes (*VvCSD1*, *VvCSD2*, *VvCSD3*, *VvFSD1*, *VvFSD2*, and *VvMSD2*) were downregulated at 24 h. Only two genes (*VvCSD3* and *VvMSD2*) were downregulated at 6 h and maintained similar levels at the following time points.

### 3.9. Coexpression Network of the VvSOD Gene Family and TF Genes

**Figure 10:**
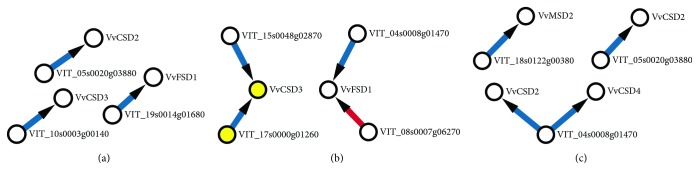
Coexpression networks of the grapevine SOD gene family and TF genes. The coexpression networks were established based on the Pearson correlation coefficients of gene pairs under cold, heat, drought, and salt stresses. All of the Pearson correlation coefficients of coexpression gene pairs were significant at the 0.05 significance level (*p* value). The different edge line colors indicated different relevance levels of coexpression gene pairs (the red color and blue color represent positive correlation and negative correlation, respectively). The arrow points from TF genes to VvSOD genes.

To determine the potential coexpression relationships between the VvSOD genes and TF genes, we conducted one coexpression network based on the microarray data under four stress treatments (*p* value < 0.05, and PCC<−0.95 or >0.95). It showed that VvSOD genes and TF genes corresponded both positively and negatively (Figure 10). A total of 11 gene pairs under three treatments (heat, salt, and drought) were correlated with each other, including five VvSODs (*VvCSD2*, *VvCSD3*, *VvCSD4*, *VvMSD2*, and *VvFSD1*) and eight TF genes (*VIT_05s0020g03880*, *VIT_19s0014g01680*, *VIT_10s0003g00140*, *VIT_17s0000g01260*, *VIT_08s0007g06270*, *VIT_04s0008g01470*, *VIT_15s0048g02870*, *VIT_18s0122g00380*). Under heat treatment, three gene pairs showed a negative correlation (Figure 10(a)). Only one gene pair was positively correlated with one another under salt stress (*VvFSD1* and *VIT_08s0007g06270*) (Figure 10(b)). Interestingly, correlated gene pair (*VvCSD3* and *VIT_17s0000g01260*, noted by yellow node) was also predicted to have regulation interaction (Table S6). Additionally, the result inferred that there was a negative correlation between *VIT_04s0008g01470* with two VvSOD genes (*VvCSD3* and *VvMSD2*) under PEG treatment (Figure 10(c)).

## 4. Discussion

With the development of second-generation sequencing technologies and the elucidation of the multiple functions of SOD genes, research into the identification of SOD genes has progressed rapidly. The SOD gene family has been identified in many species, such as *Arabidopsis* [20], longan [17], rice [39], poplar [9], banana [22], sorghum [40], tomato [8], cotton [23, 41], and cucumber [37].

In the present study, we identified 10 SOD genes in the grapevine genome that could be classified into three SOD types (six Cu/ZnSODs, two FeSODs, and two MnSODs) based on the domain and phylogenetic tree analyses. The 10 SOD genes possessed different intron numbers. A previous study reported that Cu/Zn-SODs containing different intron numbers and positions showed no exon-intron structural similarities in related species [42]. It is worth noting that one tandem pair of VvSOD genes (*VvMSD1* and *VvMSD2*) exhibited similar intron/exon organization patterns.

The promoters of VvSODs contain large amounts of light-responsive *cis*-elements that may contribute to light stress resistance. Gupta et al. detected increased resistance to oxidative stress in transgenic plants with an overexpressed Cu/Zn-SOD gene [43]. Transgenic *A. thaliana* plants overexpressing a miR398-resistant form of CSD2 accumulated more CSD2 mRNA than plants overexpressing a regular CSD2 and as a result were much more tolerant of intensive light [44]. Similar observations have also been reported in *Nicotiana tabacum* and *N. plumbaginifolia* [45, 46]. All VvSOD genes except for VvFSD2 possessed the MYB binding site. It had been reported that MYB transcription factors played regulatory roles in developmental processes and defense responses in plants [47]. The existence of abundant MYB-related *cis*-elements indicated that it may involve in the regulation of the expression of VvSOD genes.

By predicting TFs which may regulate VvSODs, several TFs were obtained (Table S6). These TFs belonged to a different TF protein family, including SBP, MYB, WRKY, ERF, CPP, LBD, TCP, HD-ZIP, AP2, and Dof family. Many of them had been found involve in a variety of biological functions like flower development, biotic and abiotic stresses, cell proliferation, hormonal response, and carbohydrate metabolism [48–54]. It has been reported that TFs regulate the target genes by both activate way and repress way [55, 56]. The coexpression results of VvSODs and TF genes also showed evidence for this conclusion. TF regulation prediction showed that *VIT_17s0000g01260* may interact with *VvCSD3*. Coexpression result identified this prediction with a negative relationship. However, the roles of TFs in VvSOD regulatory networks, as well as their interaction mechanism, remain to be fully elucidated through experimental verification and bioinformatic analysis.

Gene duplication provides new genetic material and novel genes and occurs via tandem, segmental, or genome-wide duplication [57–60]. In our study, only one segmental duplication event (*VvMSD1* and *VvMSD2*) and one tandem duplication event (*VvCSD4* and *VvCSD5*) were identified.

The expression profiles of the 10 VvSOD genes in the different tissues indicated that most of the VvSOD genes were expressed, and a few genes displayed tissue-specific expression patterns. *VvCSD4* and *VvCSD5*, identified as tandemly duplicated gene pairs, exhibited similar gene expression patterns. Moreover, *VvMSD1* and *VvMSD2*, the segmentally duplicated gene pair, showed different expression patterns in all the tested tissues. Li et al. reported that the attribution of duplication mode to the expression divergence implies a different evolutionary course of duplicated genes [61]. Adams et al. found that organ-specific and biased expression or silencing of duplicated gene pairs existed in *Gossypium hirsutum* [62]. A similar phenomenon was also reported in wheat [63].

The qRT-PCR results revealed that every VvSOD gene responded to at least one abiotic stress treatment performed in our study (Figure 9). Notably, only *VvCSD1* was activated strongly under heat stress. Moreover, *VvCSD1* also expressed highly under cold stress. This suggest that *VvCSD1* may play a vital role in the low/high temperature defense. *VvCSD6* showed the highest expression level compared with other genes under drought stress. Promoter analysis revealed that *VvCSD6* harbored 3 MYB binding sites which involved in drought inducibility, which could explain the significant expression of *VvCSD6* under drought stress. Under salt stress, only the genes *VvCSD3* and *VvCSD6* altered obviously. Taken together, the expression pattern under various stress conditions suggested different VvSOD genes could perform its own function under different stresses. This research constitutes the first comprehensive study of the SOD gene family in grapevine and may provide valuable information for elucidating the classification, evolution, and putative functions of the grapevine SOD family on the whole-genome scale.

## 5. Conclusion

In the present study, we identified 10 SOD gene grapevine, including three types (6 Cu/Zn SODs, 2 FeSODs, and 2 MnSODs), which were unevenly distributed on 12 chromosomes. Syntenic analysis revealed that *VvMSD1* and *VvMSD2* were derived from segmental duplication, and *VvCSD4* and *VvCSD5* belong to a pair of tandemly duplicated genes. Promoter analysis indicated that VvSODs contain large amounts of light-responsive *cis*-elements which may contribute to light stress resistance. Furthermore, the expression profiles of the 10 VvSOD genes in the different tissues and abiotic stresses indicate that a few genes displayed tissue-specific expression patterns and VvSODs play roles in different aspects of abiotic stress. Finally, coexpression networks of VvSODs and TF genes under the four abiotic stresses were generated based on the microarray data. *VIT_17s0000g01260* predicted to regulate *VvCSD3*, coexpression analysis verified the corelationship of these two genes. This suggested that *VIT_17s0000g01260* may participate in the regulation of *VvCSD3*. However, the regulation mechanism needs further investigation.

This was the first comprehensive study of the SOD gene family in grapevine and may provide valuable information for understanding the classification, evolution, and putative functions of the grapevine SOD family on the genome-wide scale.

## Figures and Tables

**Table 1:** Chromosome location, subcellular prediction, and physicochemical properties of grape SOD proteins.

Gene name	Gene ID	ORF length	Chromosomal localization	Molecular weight	Theoretical pI	Aliphatic index	Grand average of hydropathicity (GRAVY)	Subcellular prediction
VvCSD1	VIT_202s0025g04830.1	969	chr2(4357308 4364558)	33844.25	5.66	88.66	0.007	Cytoplasmic
VvCSD2	VIT_206s0061g00750.1	666	chr6(18276905 18281206)	22537.5	5.87	92.22	0.067	Cytoplasmic
VvCSD3	VIT_208s0007g07280.1	471	chr8(20888649 20892112)	15786.74	7.19	83.78	-0.164	Cytoplasmic
VvCSD4	VIT_214s0030g00830.1	381	chr14(5076284 5079112)	12926.4	5.33	81.19	-0.202	Cytoplasmic
VvCSD5	VIT_214s0030g00950.1	351	chr14(5248285 5250067)	12149.69	4.93	84.83	0.157	Cytoplasmic
VvCSD6	VIT_214s0036g01320.1	240	chr14(12383065 12384913)	8594.7	5.81	83.67	-0.394	Cytoplasmic
VvMSD1	VIT_206s0004g07950.1	687	chr6(8686646 8687855)	25615.29	7.14	94.56	-0.358	Mitochondrial
VvMSD2	VIT_213s0067g02990.1	852	chr13(1602822 1606910)	31456.18	9.29	86.08	-0.364	Mitochondrial
VvFSD1	VIT_210s0042g00100.1	990	chr10(12756538 12763591)	37779.23	8.62	77.11	-0.522	Chloroplast
VvFSD2	VIT_216s0013g00260.1	681	chr16(5200339 5206132)	25268.79	8.48	83.76	-0.375	Chloroplast

## Data Availability

Previously reported microarray data were used to support this study and are available at the GEO dataset (https://www.ncbi.nlm.nih.gov/geo/). The accession numbers are in the following: GSE36128, GSE31594, and GSE31675. The qRT-PCR data used to support the findings of this study are included within the supplementary information file (Table S5).

## Supplementary Materials

Supplementary 1 Table S1: the grape and Arabidopsis SOD sequence information (including the name of each sequence, SOD type, and subcellular location) which is used for phylogenetic tree analysis.

Supplementary 2 Table S2: the Ka/Ks ratios for VvSOD proteins which were calculated using MEGA 6.06.

Supplementary 3 Table S3: the specific primer for qRT-PCR of each VvSOD gene.

Supplementary 4 Table S4: the Pearson correlation coefficient (PCC) value of four treatments. Coexpressed gene pairs were filtered with a PCC cutoff of 0.95.

Supplementary 5 Table S5: the PCR raw data of four treatments.

Supplementary 6 Table S6: basic information of the 21 TF genes.
