# Supplementary material for: Genome-Wide Identification, Characterization, and Expression Analysis of the Grapevine Superoxide Dismutase (SOD) Family
Source: Int J Genomics. 2019 Feb 24;2019:7350414. doi: 10.1155/2019/7350414 (PMC6409070; doi:10.1155/2019/7350414)
Supplement: Supplementary 1 — Table S1: the grape and Arabidopsis SOD sequence information (including the name of each sequence, SOD type, and subcellular location) which is used for phylogenetic tree analysis. [file 7350414.f1.doc]

**Table S1.** SOD sequences used for phylogenetic tree analysis.

| **Name** | **Protein ID** | **Species** | **Type** | **Subcellular location** |
| --- | --- | --- | --- | --- |
| VvCSD1 | VIT_202s0025g04830.1 | *Vitis vinifera* | Cu-Zn | Cytoplasmic |
| VvCSD2 | VIT_206s0061g00750.1 | *Vitis vinifera* | Cu-Zn | Cytoplasmic |
| VvCSD3 | VIT_208s0007g07280.1 | *Vitis vinifera* | Cu-Zn | Cytoplasmic |
| VvCSD4 | VIT_214s0030g00830.1 | *Vitis vinifera* | Cu-Zn | Cytoplasmic |
| VvCSD5 | VIT_214s0030g00950.1 | *Vitis vinifera* | Cu-Zn | Cytoplasmic |
| VvCSD6 | VIT_214s0036g01320.1 | *Vitis vinifera* | Cu-Zn | Cytoplasmic |
| VvMSD1 | VIT_206s0004g07950.1 | *Vitis vinifera* | Mn | Mitochondrial |
| VvMSD2 | VIT_213s0067g02990.1 | *Vitis vinifera* | Mn | Mitochondrial |
| VvFSD1 | VIT_210s0042g00100.1 | *Vitis vinifera* | Fe | Chloroplast |
| VvFSD2 | VIT_216s0013g00260.1 | *Vitis vinifera* | Fe | Chloroplast |
|  |  |  |  |  |
| AtCSD1 | AT1G08830.1 | *Arabidopsis thaliana* | Cu-Zn | Cytoplasmic |
| AtCSD2 | AT2G28190.1 | *Arabidopsis thaliana* | Cu-Zn | Cytoplasmic |
| AtCSD3 | AT5G18100.1 | *Arabidopsis thaliana* | Cu-Zn | Cytoplasmic |
| AtMSD1 | AT3G10920.1 | *Arabidopsis thaliana* | Mn | Mitochondrial |
| AtMSD2 | AT3G56350.1 | *Arabidopsis thaliana* | Mn | Mitochondrial |
| AtFSD1 | AT4G25100.1 | *Arabidopsis thaliana* | Fe | Chloroplast |
| AtFSD2 | AT5G51100.1 | *Arabidopsis thaliana* | Fe | Chloroplast |
| AtFSD3 | AT5G23310.1 | *Arabidopsis thaliana* | Fe | Chloroplast |
