# Supplementary material for: Genome-Wide Identification, Characterization, and Expression Analysis of the Grapevine Superoxide Dismutase (SOD) Family
Source: Int J Genomics. 2019 Feb 24;2019:7350414. doi: 10.1155/2019/7350414 (PMC6409070; doi:10.1155/2019/7350414)
Supplement: Supplementary 3 — Table S3: the specific primer for qRT-PCR of each VvSOD gene. [file 7350414.f3.doc]

Supplementary Table S3 Specific primer for qRT-PCR of each VvSOD gene.

| Gene | Forward primer | Reverse primer |
| --- | --- | --- |
| VvCSD1 | GGAGCTCCTGACAGAGTTTATG | ACCGAGAACCCTGACTACTT |
| VvCSD2 | CGACTGTCTCTGTTCGGATTAC | GGATTGAAATGTGCTCCTGTTG |
| VvCSD3 | AGTGGGCAGCATTCCATT | ACCAGCATTCCCAGTTGTT |
| VvCSD4 | GATGTGACTGGGAGTCTTTCTG | TCCAGCAGGATTGAAATGAGG |
| VvCSD6 | CAGATTCCTTTCACTGGATCAAAC | TTATAGCTTACCCTTTCCAAGATCA |
| VvFSD1 | GGACGACATTCCCATCATCA | GCAGCATTCCAAGACACAAG |
| VvFSD2 | GGGAACAGAGCTAGATGGAATG | TTCATGCTTTCCCAGAAGGAG |
| VvMSD2 | GGTGGTTGAAACTACTGCAAATC | GTAATCCGGCCTCACATTCTT |
